# The SARS-CoV-2 envelope protein disrupts barrier function in an *in vitro* human blood-brain barrier model

**DOI:** 10.3389/fncel.2022.897564

**Published:** 2022-08-23

**Authors:** Jiahang Ju, Yuwen Su, You Zhou, Hui Wei, Qi Xu

**Affiliations:** ^1^State Key Laboratory of Medical Molecular Biology, Institute of Basic Medical Sciences, Chinese Academy of Medical Sciences, School of Basic Medicine Peking Union Medical College, Beijing, China; ^2^Neuroscience Center, Chinese Academy of Medical Sciences, Beijing, China

**Keywords:** COVID-19, BBB disruption, SARS-CoV-2 envelope protein, astrocyte, neuroinflammation

## Abstract

Patients with coronavirus disease 2019 (COVID-19) have been frequently reported to exhibit neurological manifestations and disruption of the blood-brain barrier (BBB). Among the risk factors for BBB breakdown, the loss of endothelial cells and pericytes has caused widespread concern. Recent studies have revealed that severe acute respiratory syndrome coronavirus 2 envelope (S2E) protein caused cell death. We tested the hypothesis that the S2E protein alone could induce BBB dysfunction. The S2E protein bound to human BBB-related cells and inhibited cell viability in a dose- and time-dependent manner. Importantly, the S2E protein disrupted barrier function in an *in vitro* BBB model composed of HCMEC/D3 (brain endothelial cell line), HBVP (brain vascular pericyte), and U87MG (astrocyte cell line) cells and suppressed the expression of major genes involved in maintaining endothelial permeability and function. In addition, the S2E protein crossed the HCMEC/D3 monolayer. The S2E protein triggered inflammatory responses in HCMEC/D3 and U87MG cells. Taken together, these results show for the first time that the S2E protein has a negative impact on the BBB. Therapies targeting the S2E protein could protect against and treat central nervous system manifestations in COVID-19 patients.

## Introduction

The coronavirus disease 2019 (COVID-19) pandemic caused by severe acute respiratory syndrome coronavirus 2 (SARS-CoV-2) is continuing to spread around the world, with more than 428 million cases and nearly 6 million deaths reported globally (WHO, 2022).

Previous work has shown that in addition to conventional respiratory symptoms, SARS-CoV-2 also causes a wide range of central nervous system (CNS) complications, including but not limited to headache, loss of taste and/or smell, hallucination, and seizure (Baig, 2020; Pezzini and Padovani, 2020). But it remains unclear whether these are a consequence of direct SARS-CoV-2 infection of the nervous system, an immune-mediated para- or post-infectious condition, or sequelae of systemic disease (Ellul et al., 2020; Iadecola et al., 2020; Mao et al., 2020; Alonso-Bellido et al., 2021). One possibility is that SARS-CoV-2 invades the brain *via* the olfactory nerves due to the presence of viral particles at the neural-mucosal interface in the olfactory mucosa in autopsied patient tissues (Meinhardt et al., 2021). Alternatively, SARS-CoV-2 may cross the blood-brain barrier (BBB), as evidenced by the detection of viral RNA in cerebrospinal fluid (Moriguchi et al., 2020). In human brain organoid models, SARS-CoV-2 can directly infect neurons and choroid plexus epithelial cells (Jacob et al., 2020). SARS-CoV-2 can also induce central neuroinflammatory effects without directly invading the brain or crossing the BBB, as proteins shed from SARS-CoV-2 and cytokines may cross the BBB to affect CNS functions (Rhea et al., 2021; Yarlagadda et al., 2021). For example, the SARS-CoV-2 spike (S2S) protein alters BBB function by triggering proinflammatory responses in brain endothelial cells *in vitro* (Buzhdygan et al., 2020). The SARS-CoV-2 envelope (S2E) protein is the smallest among the four major structural proteins (75 aa in length) (Kim et al., 2020), which makes it more permeable than the S2S protein.

In COVID-19, the S2E protein alone causes acute respiratory distress syndrome (ARDS)-like pathological damage by activating toll-like receptor 2 (TLR2) on macrophages and triggering the secretion of cytokines and chemokines (Xia et al., 2021; Zheng et al., 2021). There is an abundant expression of TLR2 in microglia in the brain, which are immunological surveillance cells in the CNS (Fiebich et al., 2018). In brain endothelial cells, the expression of TLR2 was inducible upon pathogen invasion (Faure et al., 2001). Overall, the S2E protein may play a central role in COVID-19-associated neuroinflammatory symptoms by disrupting BBB functions.

The present study aimed to assess whether the SARS-CoV-2 envelope protein negatively affects BBB functions and induces neuroinflammatory effects in an *in vitro* model of the BBB.

## Materials and methods

### Cell culture and treatment

Human immortalized cerebral microvascular endothelial cells (HCMEC/D3), human brain vascular pericytes (HBVP), and human glioblastoma cells (U87MG) were obtained from Xinyu Biotechnology Co., Ltd (Shanghai, China). All cells were maintained in a humidified incubator (37°C, 5% CO_2_). HBVP and U87MG cell lines were both cultured in Dulbecco's Modified Eagle Medium (DMEM, Thermo Fisher Scientific, USA) containing 1% MEM Non-Essential Amino Acids Solution (NEAA, Thermo Fisher Scientific, USA), 1% GlutaMAX™ (Thermo Fisher Scientific, USA), 1% Penicillin-Streptomycin (Macgene, Beijing, China), and 10% Fetal Bovine Serum (FBS, Thermo Fisher Scientific, USA). HCMEC/D3 cells were maintained in culture using Endothelial Growth Medium (Xinyu Biotechnology Co., Ltd, Shanghai, China). All cells used in the experiments were between passages 10 and 15. The S2E protein with an N-terminal GST tag and a C-terminal polyhistidine tag (ENN-C5128) was obtained from Acro Biosystems (Newark, DE, USA) and was used in this study. Cells were treated with S2E protein at a final concentration of 100 nM for 12 h. The untreated controls were treated with an equal volume of DMEM. The positive controls were treated with 5 mM H_2_O_2_ for 12 h (Lee et al., 2004; Anasooya Shaji et al., 2019).

### *In vitro* human BBB model

A triple culture model of the BBB was constructed as previously described (Hatherell et al., 2011; Fikatas et al., 2021). First, HBVP cells (2.5 × 10^4^ cells/ml) were cultured on the underside of the Transwell inserts (pore size 0.4 μm, diameter 6.5 mm) pre-coated with poly-L-Lysin (Corning, New York, USA). After 12 h, U87MG cells (1.5 × 10^4^ cells/ml) were added in the same way. At the beginning of the second day, HCMEC/D3 cells (1.5 × 10^4^ cells/ml) were seeded onto luminal side. Cells were tri-cultured for an additional 3 or 4 days in the incubator at 37°C with 5% CO_2_ until the resistance of HCMEC/D3 cells on the upper side was found to be 54.1 ± 3.3 Ω cm^2^, as previously reported (Hatherell et al., 2011).

### Measurement of TEER

The barrier integrity of the BBB model was analyzed by measuring trans-endothelial electrical resistance (TEER). TEER was measured by EVOM2 (World Precision Instruments, Sarasota, FL, United States). The TEER of blank Transwell filters containing the cell culture medium was subtracted from the measured TEER of models. For each sample, TEER was measured three times and taken the average.

### FITC-dextran permeability assay

The assay was performed, as previously described with slight modifications (Czupalla et al., 2014; Leda et al., 2019). To detect the permeability of the BBB model, Fluorescein isothiocyanate (FITC)-dextran (10 kDa, 0.5 mg/mL, Sigma-Aldrich, St. Louis, MO, USA) was added to the luminal chamber for 1 h. Samples were collected from both luminal and abluminal chambers for fluorometry analysis. The concentrations of FITC-dextran were measured at 490/520 nm (emission/excitation). The permeability (Pe) coefficient of FITC-Dextran was calculated from Pe = (A/L), where [A] is abluminal concentration, [L] is luminal concentration.

### Real-time PCR

Total RNA was extracted from cell cultures using TRIzol reagent (Invitrogen, California, USA). cDNA was synthesized with 500 ng of RNA in 20 μl reaction mix using Transcriptor High Fidelity cDNA Synthesis Kit (Roche, Basel, Switzerland) according to manufacturer's instruction. Quantitative real-time PCR was performed with FastStart Universal SYBR Green Master Mix (Roche, Basel, Switzerland) on the LightCycler 96 system (Roche, Basel, Switzerland). The primer sequences used in the study are as follows:

*GAPDH*, forward, 5′-GACAGTCAGCCGCATCTTCT-3′ and reverse, 5′-TTAAAAGCAGCCCTGGTGAC-3′.

*ZO-1*, forward, 5′-GTGTCCTACCTAATTCAACTCAT-3′ and reverse, 5′-TTACCCTGAGAATTTGATCACC-3′.

*PECAM1*, forward, 5′-GAAAGCTGTCCCTGATGCCG-3′ and reverse, 5′-GGAGCAGGGCAGGTTCATAA-3′.

*PGP*, forward, 5′-TTGATGCCGTATTCCTGGGA-3′ and reverse, 5′-TTTGACCCGCACTTCAGCTA-3′.

*SLC2A1*, forward, 5′-TGGCATCAACGCTGTCTTCT-3′, and reverse, 5′-CTAGCGCGATGGTCATGAGT-3′.

*MHC-I*, forward, 5′-AGTGGGCTACGTGGACGACA-3′, and reverse, 5′-ATGTAATCCTTGCCGTCGTA-3′.

*IL-6*, forward, 5′-CAATGAGGAGACTTGCCTG-3′, and reverse, 5′-GTACTCATCTGCACAGCTCT-3′.

*IL-1*β, forward, 5′-TTCATTGCTCAAGTGTCTGAAG-3′, and reverse, 5′-AGTCATCCTCATTGCCACTGT-3′.

The *GAPDH* gene was used as the housekeeping gene to normalize the target gene expression. Relative mRNA levels were calculated using the 2^−ΔΔCt^ formula.

### Immunofluorescence staining and imaging

At experimental endpoints, specimens were washed with PBS and then fixed in 4% paraformaldehyde (pH 7.4) in PBS for 20 min at room temperature (RT). Blocking and permeabilization were performed with 5% donkey serum and 0.1% Triton X-100 in PBS for 30 min at RT. Specimens were incubated overnight at 4°C with the following primary antibodies: Rabbit anti-SARS-CoV-2 envelope protein (1:200, Cat #28904-1-AP, Proteintech, Wuhan, China), Rabbit anti-ZO-1 (1:100, Cat # 21773-1-AP, Proteintech, Wuhan, China), Rabbit anti-MHC class I (1:200, Cat # ab134189, Abcam, Cambridge, UK), Mouse anti-His-tag (1:200, Cat #D291-3, MBL, Aichi, Japan) in 5% donkey serum in PBS. Thereafter, Specimens were washed with PBS and incubated with secondary antibodies at 1:500 dilution for 1 h at RT. All secondary antibodies (Donkey anti-rabbit Alexa Fluor 488, Cat #ab150073 and Donkey anti-mouse Alexa Fluor 594, Cat #ab150108) were obtained from Abcam (Cambridge, UK). Nuclei were counterstained with *4',6-diamidino-2-phenylindole* (DAPI, 1:5000, Sigma-Aldrich, MBD0015, St. Louis, MO, USA). To stain the plasma membrane (Majkowski et al., 2012; Su et al., 2016), cells were incubated with 1,1′-dioctadecyl-3,3,3′,3′-tetramethylindodicarbocyanine, 4-chlorobenzenesulfonate salt (DiD, Thermo Fisher Scientific, Cat #D7757, USA) for 10 min at 37°C. For imaging, all sections were randomly captured at least 5 fields for each sample under 40× magnification using a Leica TCS SP8 STED 3X Super-Resolution Confocal Microscope (Wetzlar, Germany) and analyzed using the Leica LAS X software.

### Cell viability assay

Cell viability assays were evaluated using cell counting kit-8 (CCK8, MCE, New Jersey, USA) according to the manufacturer's instruction. Briefly, Cells (1 × 10^4^ cells/ml) were seeded into 96-well plates and then incubated for 24 h. After being treated with various concentrations (0–200 nM) of S2E protein for 12 h or treated with 100 nM S2E protein for indicated times (0–24 h), 10 μl of CCK8 solution was added to each well for 12 h. Absorbance was detected at 450 nm using a fluorescence plate reader (BioTek Synergy H1, Winooski, USA).

### Lactate dehydrogenase (LDH) assay

The cytotoxicity of S2E protein was quantified by measuring the amounts of LDH, which was evaluated using LDH-Glo™ cytotoxicity assay (Promega, Germany), following the manufacturer's instruction. LDH release (cytotoxicity %) was calculated by dividing the value by the maximum value. Cells were treated with 0.2% Triton X-100 for 12 h to induce nearly complete cell damage as a positive control of cell death.

### Cytokine assay by ELISA

The culture medium was collected. IL-6 and IL-1β were measured with an ELISA kit (Elabscience Biotechnology Co., Ltd, Wuhan, China) according to the manufacturer's instruction.

### Data analysis and statistics

Data were shown as mean ± standard deviation (SD) of three independent experiments. All data obtained were analyzed by Graphpad Prism version 8.4.3 (GraphPad Software, Inc.) using Student's *t*-test or one-way ANOVA with Tukey *post-hoc* tests as appropriate. Statistical significance was set at a value of *p* < 0.05.

## Results

### SARS-CoV-2 envelope protein inhibits cell viability *in vitro*

**Figure 1:**
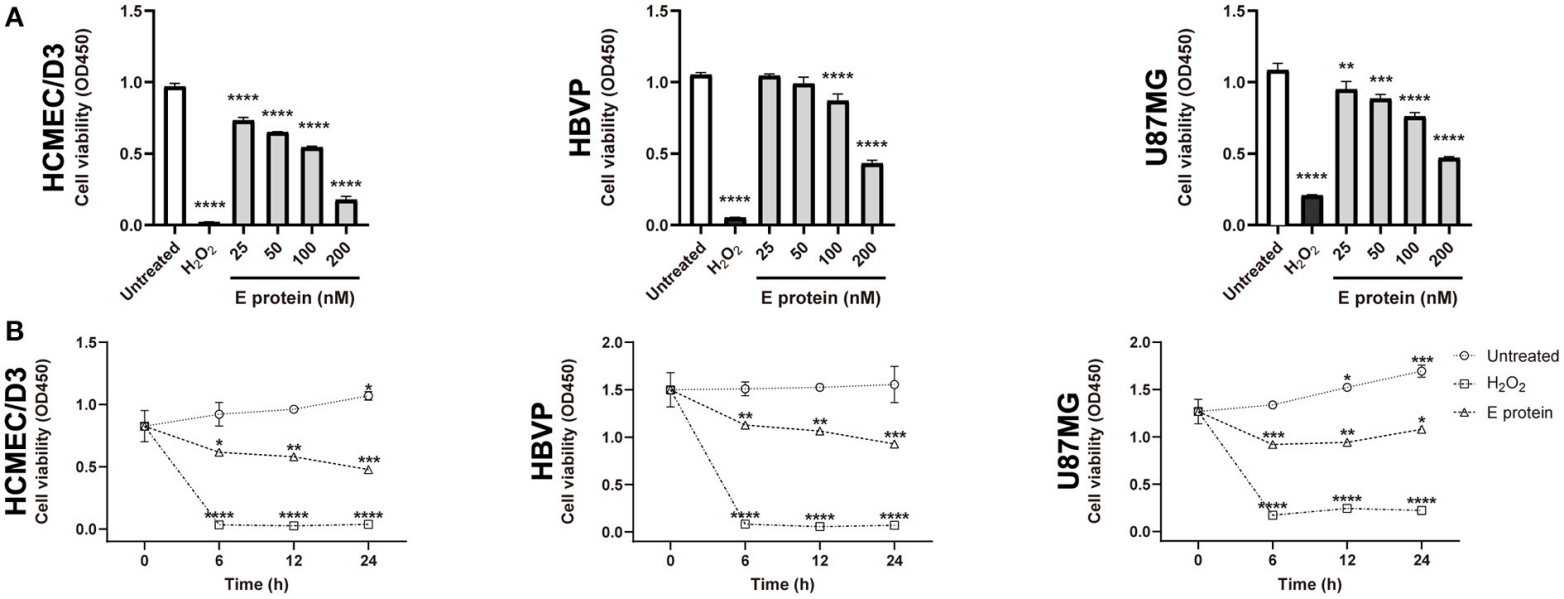
SARS-CoV-2 envelope protein affects cell viability *in vitro*. **(A)** The viability of cells after exposure to a range of S2E protein concentrations for 12 h. A CCK-8 assay was performed to assess cell viability. OD, optical density. **(B)** Time courses of the viability of HCMEC/D3, HBVP, and U87MG cells treated with 100 nM S2E protein. The data are represented as the mean ± SD of three independent experiments. *p < 0.05, **p < 0.01, ***p < 0.001, ****p < 0.0001.

The effects of the S2E protein on the proliferation of human BBB-related cell lines were assessed using a CCK-8 assay. After 12 h treatment of S2E protein, a minimum dose of 25 nM significantly decreased the viability of HCMEC/D3 cells (75.50%) and U87MG cells (87.52%), while a minimum dose of 100 nM significantly decreased the viability of HBVP cells (82.58%). At the maximum dose of 200 nM, all cell viabilities dropped below 50% (Figure 1A). There was also a time-dependent effect of the S2E protein compared to the time point 0 h of untreated controls. At a dose of 100 nM, cell viability significantly decreased as early as 6 h post-treatment (hpt), continued to decrease until 24 hpt, and finally reached 50.63% in HCMEC/D3 cells, 60.94% in HBVP cells, and 74.24% in U87MG cells (Figure 1B). Further, we evaluated the cytotoxicity of S2E protein in three cell lines using LDH assay. S2E protein resulted in 75.46% cytotoxicity of HCMEC/D3 cells. S2E protein did not affect the rate of cell death of HBVP cells and U87MG cells significantly (Supplementary Figure 1). The S2E protein colocalized with the cell membrane in all cell lines, as shown by immunofluorescent staining, suggesting that the S2E protein could bind to human BBB-related cell lines (Supplementary Figure 2).

### SARS-CoV-2 envelope protein crosses the BBB and disrupts barrier integrity and permeability

**Figure 2:**
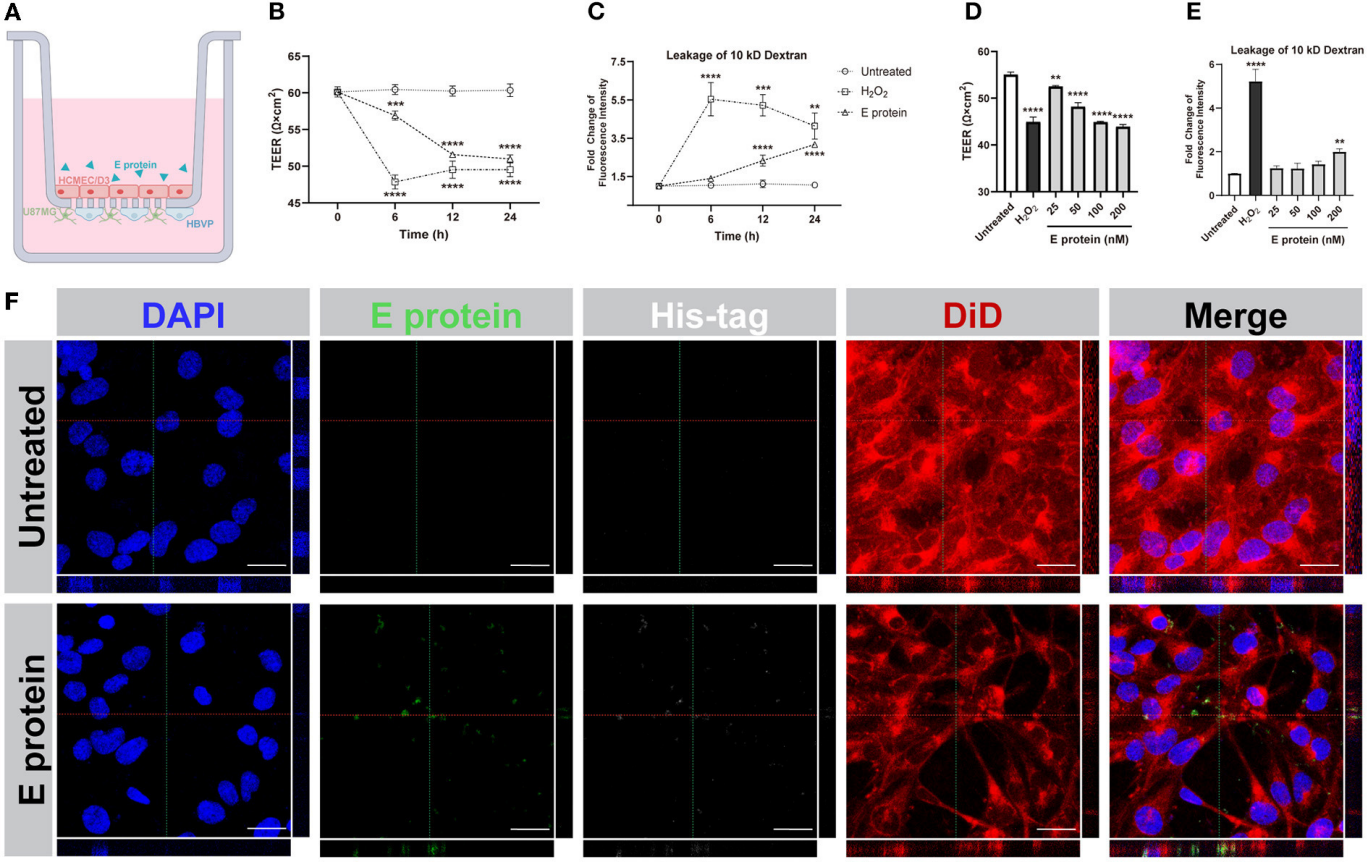
SARS-CoV-2 envelope protein crosses and impairs the BBB model. **(A)** Schematic showing the various components of the *in vitro* BBB model. **(B)** Time courses of TEER values from HCMEC/D3 monolayers exposed to the S2E protein (100 nM). **(C)** Analysis of the permeability of HCMEC/D3 monolayers treated with 100 nM S2E protein for various times. **(D)** TEER values of HCMEC/D3 monolayers treated with different S2E protein concentrations for 12 h. **(E)** HCMEC/D3 monolayer permeability after exposure to a range of S2E protein concentrations for 12 h. **(F)** Confocal top-down and cross-sectional views of the HCMEC/D3 monolayer. Scale bar = 20 μm. The data shown are the mean ± SD of three independent experiments. **p < 0.01, ***p < 0.001, ****p < 0.0001.

To evaluate the effects of the S2E protein on BBB functions, an *in vitro* Transwell barrier BBB model was established, in which HCMEC/D3 cells on the luminal side served as brain endothelial cells, while HBVP cells and U87MG cells on the underside of the insert served as brain vascular pericytes and astrocytes, respectively (Figure 2A). The S2E protein was administered on the luminal side. Then, BBB integrity was assessed by TEER, and BBB permeability was assessed by the transmissivity of 10 kDa FITC-dextran. In response to 100 nM S2E protein, the TEER values decreased in a time-dependent manner to 94.39, 85.57, and 84.57% of the baseline values at 6, 12, and 24 hpt, respectively (Figure 2B); FITC-dextran transmissivity increased to 1.31-fold, 2.19-fold, and 2.98-fold of the baseline values vs the 0 h time point of untreated controls (Figure 2C). There were also dose-dependent fluctuations in TEER and FITC-dextran transmissivity as the S2E protein concentration increased (25–200 nM) at 12 hpt (Figures 2D,E). Confocal imaging further revealed that the S2E protein bound to and passed through the HCMEC/D3 monolayer (Figure 2F).

**Figure 3:**
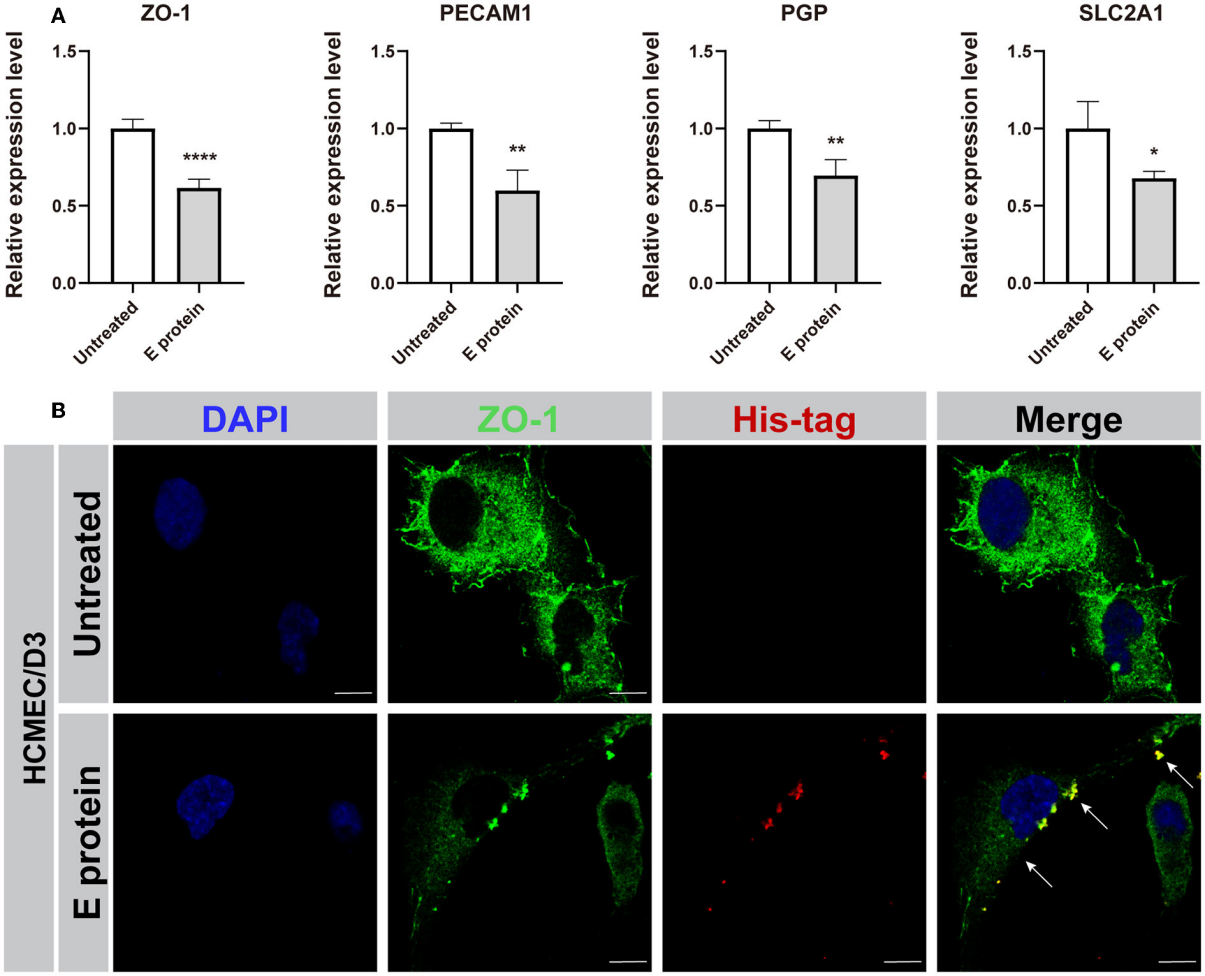
SARS-CoV-2 envelope protein impairs endothelial cell barrier function. **(A)** Relative mRNA levels of ZO-1, PECAM1, PGP, and SLC2A1 in HCMEC/D3 cells, as determined by RT-PCR. **(B)** Immunofluorescence images of ZO-1 in HCMEC/D3 cells. Scale bar = 10 μm. The data shown are the mean ± SD of three independent experiments. *p < 0.05, **p < 0.01, ****p < 0.0001.

Moreover, we explored the effect of S2E protein on endothelial cell barrier function. The expression levels of the BBB-related genes *ZO-1, PECAM1, PGP*, and *SLC2A1* were decreased in HCMEC/D3 cells at 12 hpt (Figure 3A). Furthermore, immunofluorescence staining identified the colocalization of S2E protein and ZO-1 at cell boundaries, and the ZO-1 protein was less identified and with a discontinuous pattern, suggesting disruption of this tight junction (TJ)-related gene by the S2E protein (Figure 3B). Taken together, our data strongly suggest that the S2E protein has the potential to cause BBB leakage.

### SARS-CoV-2 envelope protein triggers an inflammatory response in HCMEC/D3 and U87MG cells

**Figure 4:**
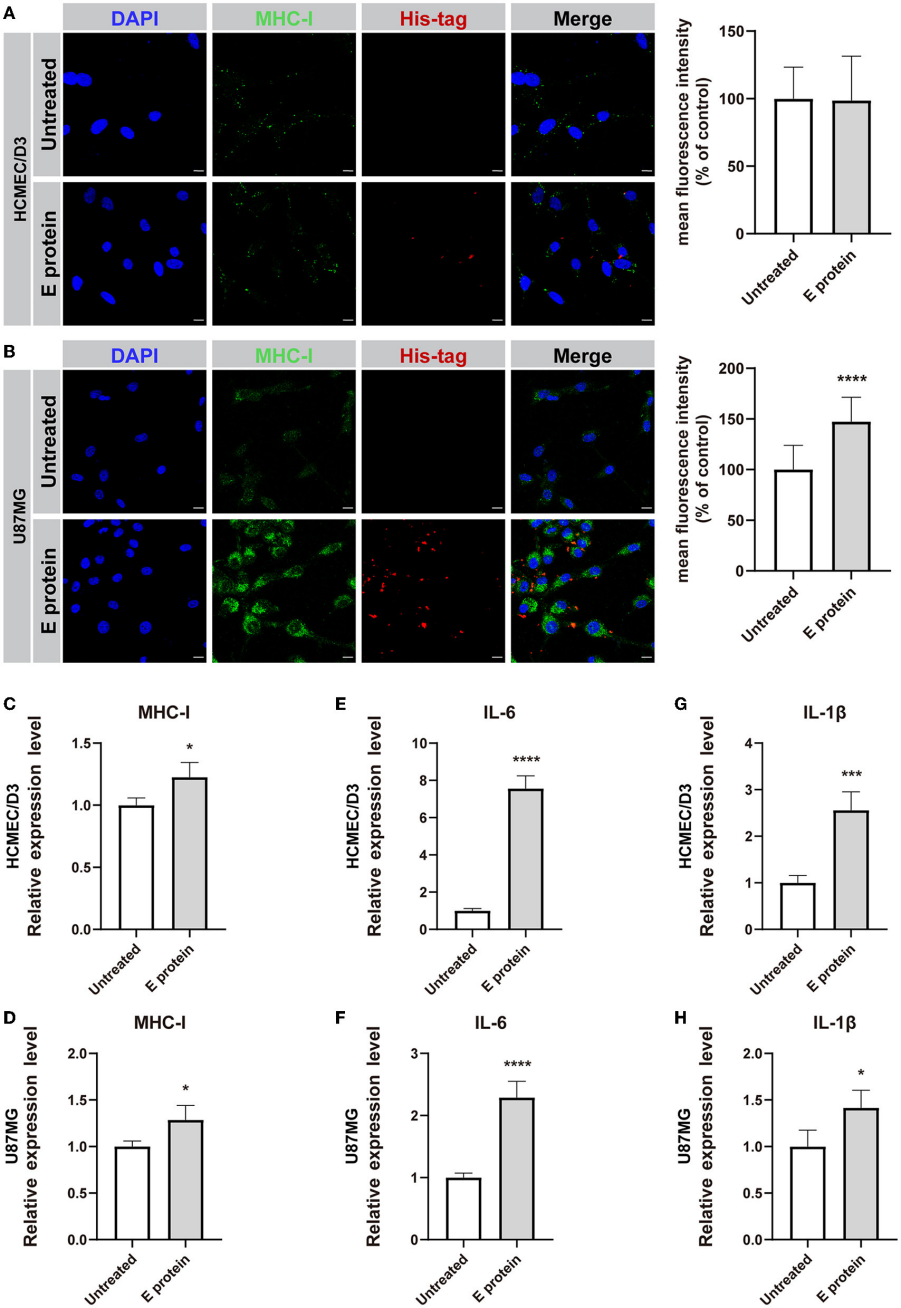
SARS-CoV-2 envelope protein induces inflammation in HCMEC/D3 and U87MG cells. **(A,B)** Immunofluorescence staining and semiquantitative analysis of MHC-I. Scale bar = 10 μm. **(C–H)** Relative mRNA levels of *MHC-I, IL-6*, and *IL-1*β, as determined by RT-PCR. The data are shown as the mean ± SD of three independent experiments. *p < 0.05, ***p < 0.001, ****p < 0.0001.

To assess whether the S2E protein triggers proinflammatory responses in cultured HCMEC/D3 and U87MG cells, the expression levels of *MHC-I, IL-6*, and *IL-1*β were assessed. Immunofluorescence staining suggested that upon S2E protein exposure, MHC class I (MHC-I) molecule expression increased in U87MG cells but not in HCMEC/D3 cells (Figures 4A,B). However, there were increased mRNA levels of *MHC-I, IL-6*, and *IL-1*β in both cell lines compared with those in the untreated group (Figures 4C–H). Furthermore, treating both cell lines with S2E protein, ELISA assay showed markedly increases only in IL-6 levels of HCMEC/D3 cells (Supplementary Figure 3).

## Discussion

The widespread neurological complications and sequelae in patients with COVID-19 (Erickson et al., 2021) indicate that SARS-CoV-2 itself or its products can trigger CNS damage. It is unclear whether these effects are directly induced by the virus or viral products (Trypsteen et al., 2020). It has been reported that the S2E protein, which affects ion channels, attacks host cells, and triggers inflammation as a virulence factor (Xia et al., 2021). In this study, we examined the ability of the S2E protein to cross the BBB by disrupting the endothelial barrier and triggering inflammatory responses in glial cells in an *in vitro* BBB model. The S2E protein was able to reduce viability in all three BBB-related cell lines, disrupt BBB integrity and the expression levels of BBB-specific genes, and trigger BBB inflammation.

The S2E protein forms viroporins on the plasma membrane of host cells, which have ion channel activities and are permeable to cations, as reported in other coronaviruses (Schoeman and Fielding, 2019). The massive efflux of intracellular ions leads to cell shrinkage and stimulates the apoptotic cascade by disrupting the negative transmembrane potential (Kunzelmann, 2016). Viroporins can further induce endoplasmic reticulum (ER) stress by inhibiting ER folding capacity, delaying glycoprotein trafficking or membrane remodelling, and ultimately initiating the apoptosis cascade (Fung et al., 2015). Viroporins also stimulate pyroptosis through inflammasome activation (Guo et al., 2015). Pyroptosis is characterized by the formation of pores in the plasma membrane, resulting in the influx/efflux of ions that ultimately leads to cell lysis and the release of intracellular inflammatory substances (Bergsbaken et al., 2009). In CNS pathological conditions such as Parkinson's disease, Alzheimer's disease, and HIV-associated neurocognitive disorder (HAND), brain cells undergo pyroptosis, causing neuroinflammation and neurodegeneration (McKenzie et al., 2020). For example, in HAND, the HIV-1 envelope protein gp120 is required for the formation of the NLRP3 inflammasome prior to the initiation of NLRP3-dependent pyroptosis and IL-1β production in microglia (He et al., 2020). Similarly, the S2E protein was able to induce Nlrp3 expression by activating TLR2 on macrophages (Zheng et al., 2021). TLR2-dependent signalling induced the production of proinflammatory cytokines during coronavirus infection independent of viral entry (Zheng et al., 2021).

The S2E protein disrupted BBB tight junctions by negatively affecting the expression level and subcellular distribution of the TJ protein ZO-1. After S2E protein challenge, *ZO-1* mRNA levels were decreased in HCMEC/D3 cells, and less well-defined and discontinuous distribution of ZO-1 protein was observed at cell boundaries. ZO proteins belong to the family of membrane-associated guanylate kinases, which have several conserved domains, including N-terminal PDZ, SH3, and GUK domains, as well as a C-terminal actin filament binding site (Fanning et al., 1998; Itoh et al., 1999). ZO proteins are key scaffolds that bridge components of the TJ complex and are key indicators of BBB integrity (Wolburg and Lippoldt, 2002; Shin and Margolis, 2006). It has been reported that the S2E protein can interact with the PDZ domain of ZO-1 on host cells, which disrupts the dimerization of ZO proteins or the aggregation of TJ proteins (such as occludins and claudins) into an intact barrier (Utepbergenov et al., 2006; Rodgers et al., 2013; Shepley-McTaggart et al., 2021). In addition, the S2E protein may facilitate the endocytosis of ZO-1 in a PDZ-binding motif-dependent manner by hijacking the binding sites for PLAZ1 and connexin 43 (Giepmans and Moolenaar, 1998; Teoh et al., 2010).

The S2E protein may trigger CNS inflammatory responses by activating astrocytes when crossing the leaky BBB. In viral infection, viral proteins can be sensed by MHC-I molecules on immune cells and further initiate antigen presentation cascades (Croft et al., 2019). Moreover, MHC-I is widely expressed in CNS neurons and glial cells (Janeway et al., 2001). *In vivo* and *in vitro* evidence suggested that MHC-I expression was upregulated in astrocytes following systemic immune activation by the administration of polyinosinic-polycytidylic acid (Sobue et al., 2018; Li et al., 2021). The expression of astrocytic MHC-I in the brain significantly activated microglial cells, reduced dendritic spine density, and impaired sociability and recognition memory in mice (Sobue et al., 2018). It is possible that overreactive astrocytes enhanced synaptic pruning in neurons (Oliveira et al., 2004; Freria et al., 2010). In contrast, inhibition of MHC-I in astrocytes stabilized synapses and diminished astrogliosis (Scorisa et al., 2011; Bombeiro et al., 2017). The present study showed that S2E protein administration increased the mRNA levels of *MHC-I, IL-6*, and *IL-1*β in U87MG astroglia, suggesting the occurrence of CNS inflammation. Thus, it is important to keep in mind that the hazard of the COVID-19 pandemic is not only the SARS-CoV-2 virus itself and that multisystem inflammatory damage caused by viral proteins may cause broad and lasting side effects.

**Figure 5:**
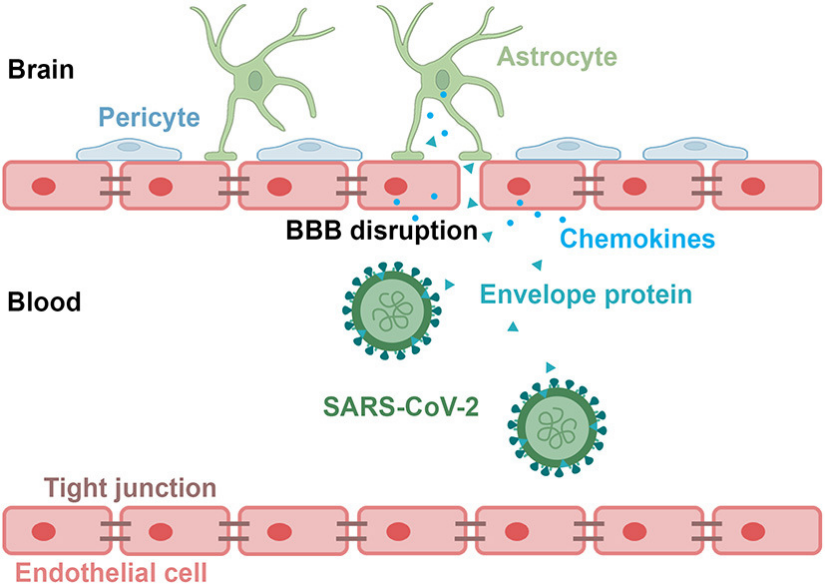
A schematic diagram depicting the potential mechanism by which the SARS-CoV-2 envelope protein crosses the BBB. The S2E protein disrupts BBB integrity, leading to inflammation in endothelial cells and astrocytes.

The possible mechanism by which the S2E protein crosses the BBB is shown in Figure 5. During infection, the envelope protein can bind to brain endothelial cells and traverse the BBB, leading to damaging the BBB and inducing inflammatory responses in astrocytes. In addition, the inflammatory factors produced by brain endothelial cells and astrocytes, in turn, may exacerbate BBB damage.

This study has several limitations. First, the *in vitro* BBB model used in this study lacks further microglia and neuronal interactions. The breakdown of the BBB can induce microglial activation and trigger CNS inflammation (da Fonseca et al., 2014; Bowman et al., 2018). Evidence suggests that overactivated microglia induce abnormal axonal dendritic pruning and even neuronal demyelination, ultimately leading to behavioural sickness (Harry, 2013). An *in vivo* S2E protein challenge model is required in future studies. Second, there was a dose accumulation effect of the S2E protein in our study, suggesting an association between S2E protein load and BBB breakdown. Even though the S2E protein has been detected in multiple tissues of the human body (such as pharyngeal discharge, lung tissues, microvascular endothelial cells, and cerebrospinal fluid), there is a lack of sufficient information on the S2E protein load, as well as that of other viral proteins, in patients with COVID-19 (Benameur et al., 2020; Nuovo et al., 2020; Magro et al., 2021). More importantly, the relationship between S2E protein load and SARS-CoV-2 viral load, as well as its relationship with COVID-19-related neuropsychiatric complications, remains unclear. Therefore, in future studies, it will be necessary to measure the viral protein load in these patients.

In conclusion, our study indicated that the SARS-CoV-2 envelope protein could bind to brain endothelial cells and cross the BBB, leading to neuroinflammation. Our findings improve the understanding of the mechanisms of SARS-CoV-2 invasion in the CNS and suggest that blocking the SARS-CoV-2 envelope protein could be an important therapeutic strategy for the neurological complications of COVID-19 patients.

## Data availability statement

The original contributions presented in the study are included in the article/Supplementary material, further inquiries can be directed to the corresponding author/s.

## Author contributions

JJ performed the main experiments, analyzed the data, and drafted the manuscript. YS and YZ set up the human *in vitro* BBB model. QX and HW conceived of the study, participated in its design, and revised the manuscript. All authors contributed to the article and approved the submitted version.

## Funding

This work was supported by research grants from the National Key Research and Development Program of China (2020YFA0804502), the Ministry of Science and Technology of the People's Republic of China (2021ZD0203001, 2022ZD0211700, 2021ZD0200600, and 2021ZD0202000), the National Natural Science Foundation of China (82071504, 81930104, 82171447, and 81871011), CAMS Innovation Fund for Medical Sciences (2021-I2M-1-020), and Science and Technology Program of Guangdong (2018B030334001).

## Conflict of interest

The authors declare that the research was conducted in the absence of any commercial or financial relationships that could be construed as a potential conflict of interest.

## Publisher's note

All claims expressed in this article are solely those of the authors and do not necessarily represent those of their affiliated organizations, or those of the publisher, the editors and the reviewers. Any product that may be evaluated in this article, or claim that may be made by its manufacturer, is not guaranteed or endorsed by the publisher.

## Abbreviations

COVID-19: coronavirus disease 2019
BBB: blood-brain barrier
SARS-CoV-2: severe acute respiratory syndrome coronavirus 2
S2S: SARS-CoV-2 spike
S2E: SARS-CoV-2 envelope
CNS: central nervous system
ARDS: alone causes acute respiratory distress syndrome
TLR2: toll-like receptor 2
MHC-I: MHC class I
ER: endoplasmic reticulum
HAND: HIV-associated neurocognitive disorder.
