# Supplementary material for: The SARS-CoV-2 envelope protein disrupts barrier function in an in vitro human blood-brain barrier model
Source: Front Cell Neurosci. 2022 Aug 23;16:897564. doi: 10.3389/fncel.2022.897564 (PMC9445123; doi:10.3389/fncel.2022.897564)
Supplement: Supplementary file 1 [file Data_Sheet_1.docx]

**
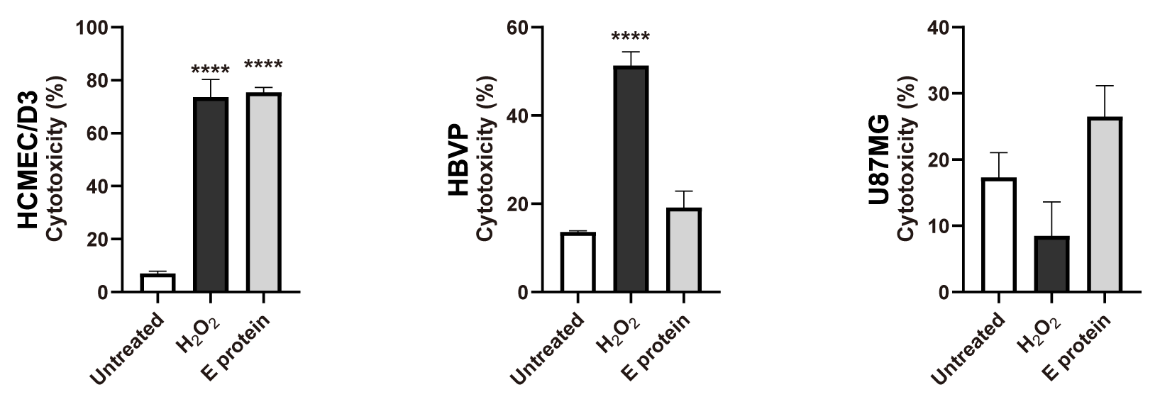
**

**Supplementary Figure 1.** The cytotoxicity of S2E protein was quantified by the LDH release into the culture media. The data are shown as the mean ± SD of three independent experiments. *****p* < 0.0001.

**
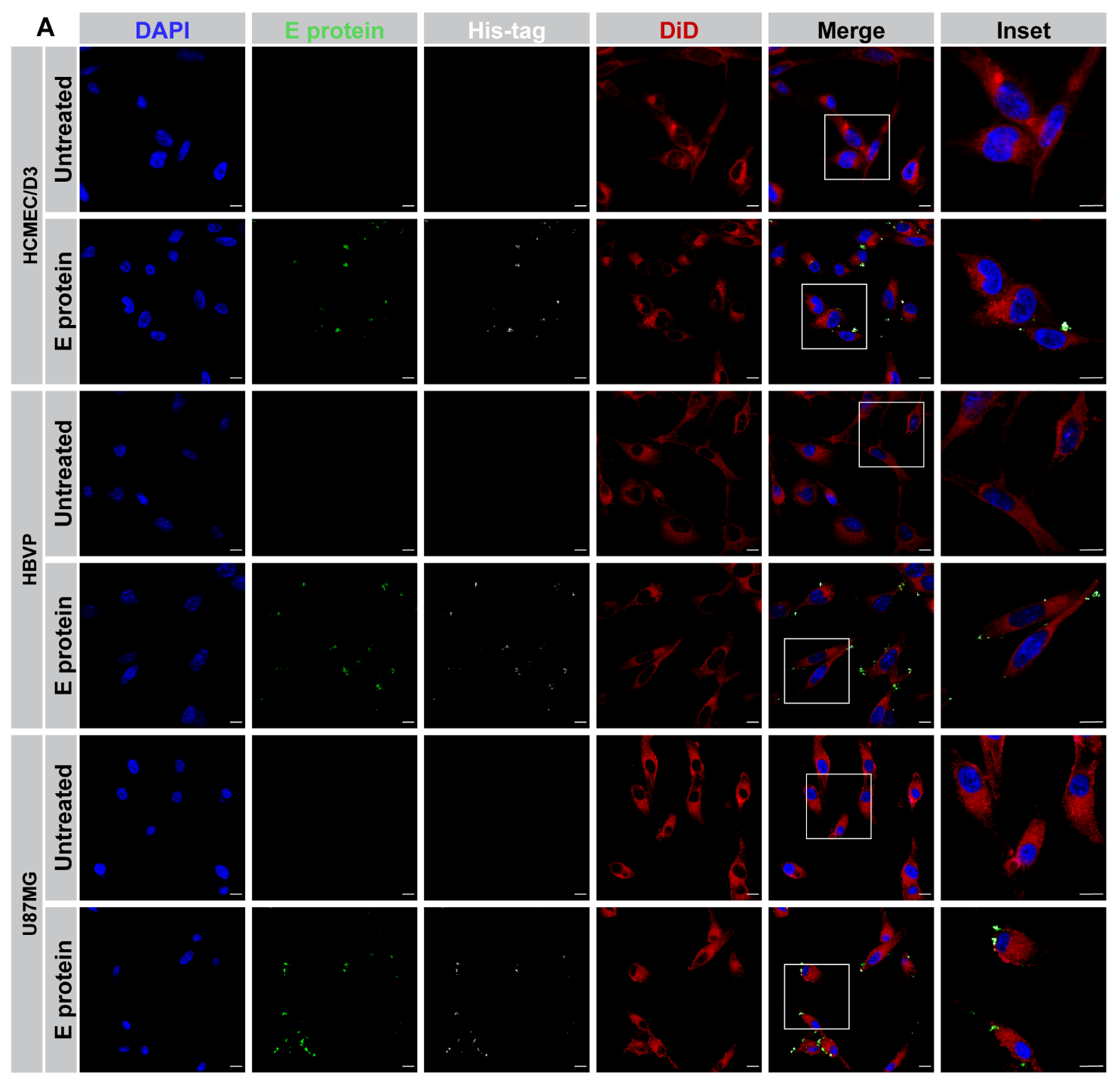
**

**Supplementary Figure 2.** Confocal images of cells exposed to S2E protein (100 nM) for 12 h. Scale bar = 10 µm. The data are representative of three independent experiments.

**
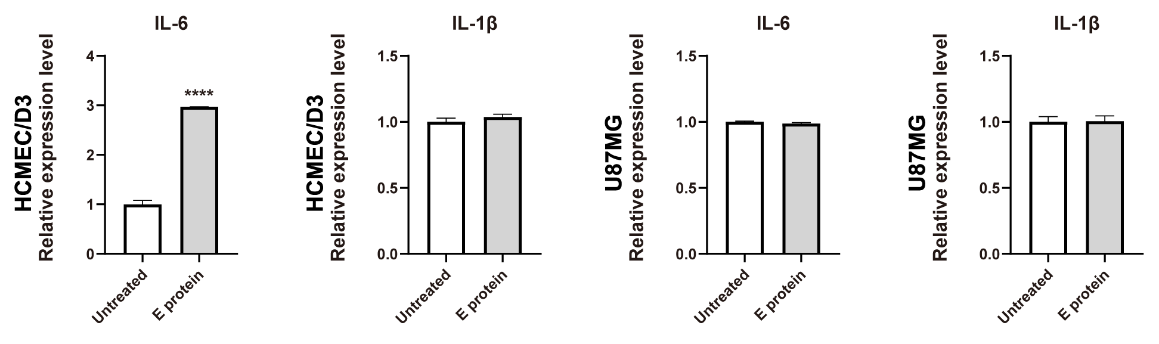
**

**Supplementary Figure 3.** Relative expression levels of IL-6 and IL-1β were detected by ELISA. The data are shown as the mean ± SD of three independent experiments. *****p* < 0.0001.
